# EHMTI-0275. Headache and cervical spine disturbances in a patient with osteoarthritis of temporomandibular joint – one-year-follow-up

**DOI:** 10.1186/1129-2377-15-S1-C5

**Published:** 2014-09-18

**Authors:** T Badel, V Basic Kes, D Rosic, D Zadravec, J Kern

**Affiliations:** 1 Department of Removable Prosthodontics, School of Dental Medicine, Zagreb, Croatia; 2 Department of Neurology, Clinical Hospital Center Sestre milosrdnice, Zagreb, Croatia; 3 Department for Rheumatic Disease, Center for Rheumatic Disease Physical Therapy and Rehabilitation "dr Drago Cop", Zagreb, Croatia; 4 Department of Diagnostic and Interventional Radiology, linical Hospital Center Sestre milosrdnice and Rehabilitation "dr Drago Cop", Zagreb, Croatia; 5 Department of Medical Statistics Epidemiology and Medical Informatics, School of Public Health "Andrija Štampar", Zagreb, Croatia

## Introduction

Temporomandibular joint (TMJ)-related pain also includes the craniomandibular and cervicocranial areas due to the topographic-functional closeness.

## Aims

To determine the relationship between types of headaches and cervical spine (CS) disorders in a patients with osteoarthritis (OA) of TMJ with one-year-follow-up.

## Methods

65 patients (mean age 47.4, 95.4% women) were consecutively treated for signs and symptoms of OA of TMJ. A definitive diagnosis of OA was confirmed by magnetic resonance imaging. The patients were examined by a dentist, a neurologist, and a physiatrist-rheumatologist. Pain intensity in TMJ was shown on the visual-analogue scale (VAS 0-10). They were treated by an occlusal splint and physical therapy with one-year-follow-up.

## Results

The applied treatment modalities achieved a significant reduction of pain (p<0.001) in the TMJ at first examination and one-year-follow-up (mean values on VAS: 6.58 and 1.67). 46.2% of patients did not have a CS diagnosis and 53.9% of patients did not have headaches. 16.9% of them had migraines, 23.1% had CS-related headache and 6.1% of patients had tension-types headaches. Cervical syndrome was found in 10.8% of patients. 26.1% had cervicobrachial syndrome, 7.7% had cervicocephalic syndrome and 9.2% of patients had both. The SC syndrome was significant regarding the patients’ age (mean age 40.8 as opposed to 53.1 years, with p=0.0002), whereas there were no differences for headaches.

## Conclusions

The relationship of SC disturbances with the higher age of patients was determined in patients with OA of TMJ. The existence of comorbidity with headaches does not affect treatment success of TMJ.

No conflict of interest.

